# Successful Laparoscopic Management of Non-communicating Rudimentary Horn Pregnancy

**DOI:** 10.7759/cureus.27268

**Published:** 2022-07-26

**Authors:** Maen M Alrawashdeh, Fawaz Alkazaleh

**Affiliations:** 1 School of Medicine, Royal College of Surgeons in Ireland, Dublin, IRL; 2 Faculty of Medicine, The University of Jordan, Amman, JOR

**Keywords:** laparoscopic management, rudimentary horn pregnancy, prenatal ultrasound, ectopic pregnancy, mullerian duct anomaly

## Abstract

Unicornuate uteri are a type of Mullerian duct anomaly and the majority present with rudimentary horns. Rudimentary horn pregnancies are extremely rare and have a high risk of rupture. A high index of suspicion is needed to diagnose them early and unfortunately, the majority of cases are undetected until the patient presents with a ruptured uterus. Early diagnosis and management will reduce morbidities and mortality for patients.

We present a case of a 29 year old who had a routine ultrasound scan in the first trimester that raised an index of suspicion for a rudimentary horn pregnancy. An MRI scan was performed and supplemented the ultrasound findings. The patient underwent laparoscopic management, and the non-communicating rudimentary horn, the foetus, and the attached tube were excised. The patient had a smooth recovery and had no complications.

Due to the rarity of rudimentary horn pregnancies, a high index of suspicion is needed for a diagnosis. Timely detection and intervention are crucial to prevent complications. Ultrasound scans and MRIs can aid in the diagnosis. Traditional management involved laparotomy, but with surgical advancements, laparoscopic surgery can be utilized as a less invasive alternative.

## Introduction

A non-communicating rudimentary horn pregnancy (RHP) is an extremely rare event with a reported incidence of 1 in 76,000 - 150,000 pregnancies [1]. It is proposed that non- communicating RHPs occur because of transperitoneal migration of sperm to the contralateral unicornuate uterus [2]. The key point is early detection because this condition if left untreated may present a stormy situation, especially in the second trimester [3]. In addition, the fetal outcomes are poor, with only a 13% survival rate according to certain reports [4]. Timely diagnosis and treatment of RHPs are very crucial to prevent devastating maternal outcomes. Despite this, the majority of the cases have a late diagnosis and a poorer outcome [5].

We report this rare case of non-communicating rudimentary horn pregnancy in a young lady who was diagnosed early and proper surgical strategy was applied. The present case depicts the high importance of early diagnosis since it allows safer treatment compared to an intervention on an acutely ill patient.

## Case presentation

**Figure 1:**
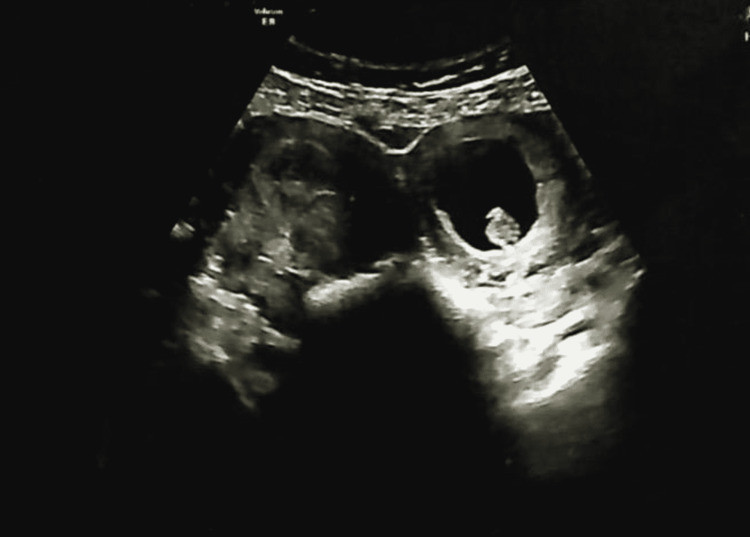
Ultrasound scan showing foetus in the extra uterine space, raising an index of suspicion for an RHP RHP: rudimentary horn pregnancy

**Figure 2:**
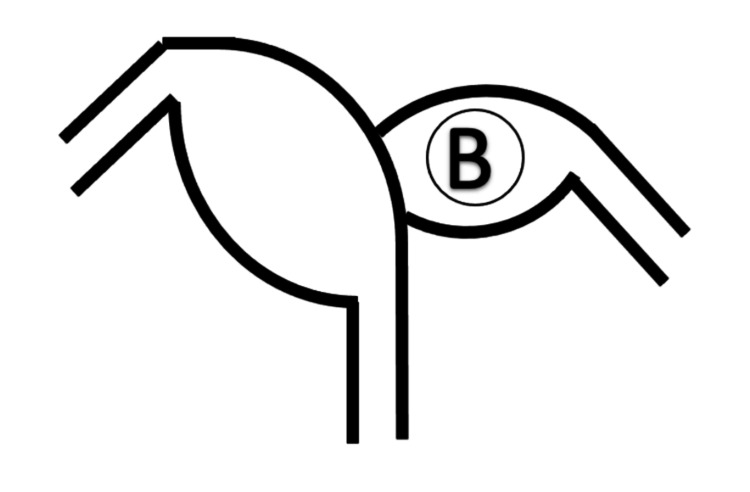
Drawing showing a non-communicating rudimentary horn pregnancy with (B) representing the fetus

A 29-year-old woman, Gravida 1 Para 0, not known to have any past medical or surgical history, came in for a routine antenatal check-up during her 10th gestational week. Up to this point, she had no complaints, and all her vitals were normal, however, upon ultrasound examination, an empty uterus with a thickened endometrium was seen and a viable 10-week foetus was visualized in an extra-uterine space surrounded by thick-walled myometrium (Figure 1). This raised a high index of suspicion for the diagnosis of a rudimentary horn with an associated ectopic pregnancy (Figure 2), so an MRI was requested to confirm our diagnosis.

The MRI scan showed a prominent uterine body that appeared dextrorotated and a significantly thickened endometrial lining measuring 2.5 cm in thickness. Furthermore, there was a fluid-filled rounded structure with a diffusely thick wall seen on the left side of the pelvis measuring 4.4 x 4.6 cm in cross-section and appeared to be connected to the uterine body at its lower segment. This appearance was highly suggestive of a gestational sac in a rudimentary uterine horn. It is also important to note that no renal abnormalities were detected in this patient.

**Figure 3:**
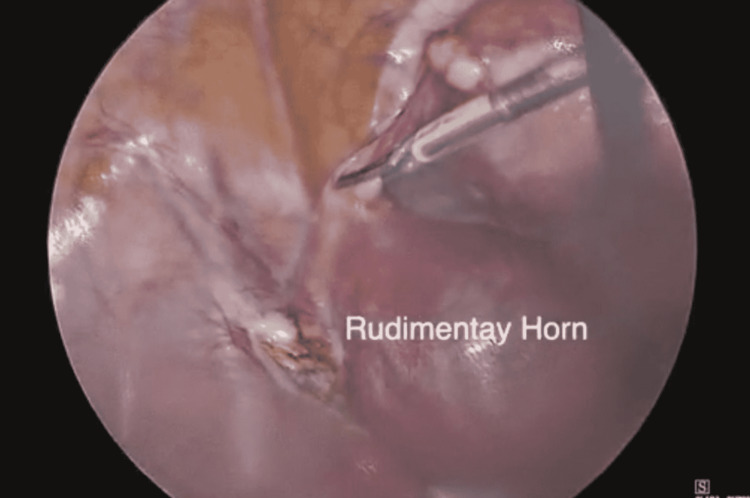
Intact left non-communicating rudimentary horn can be seen laparoscopically

**Figure 4:**
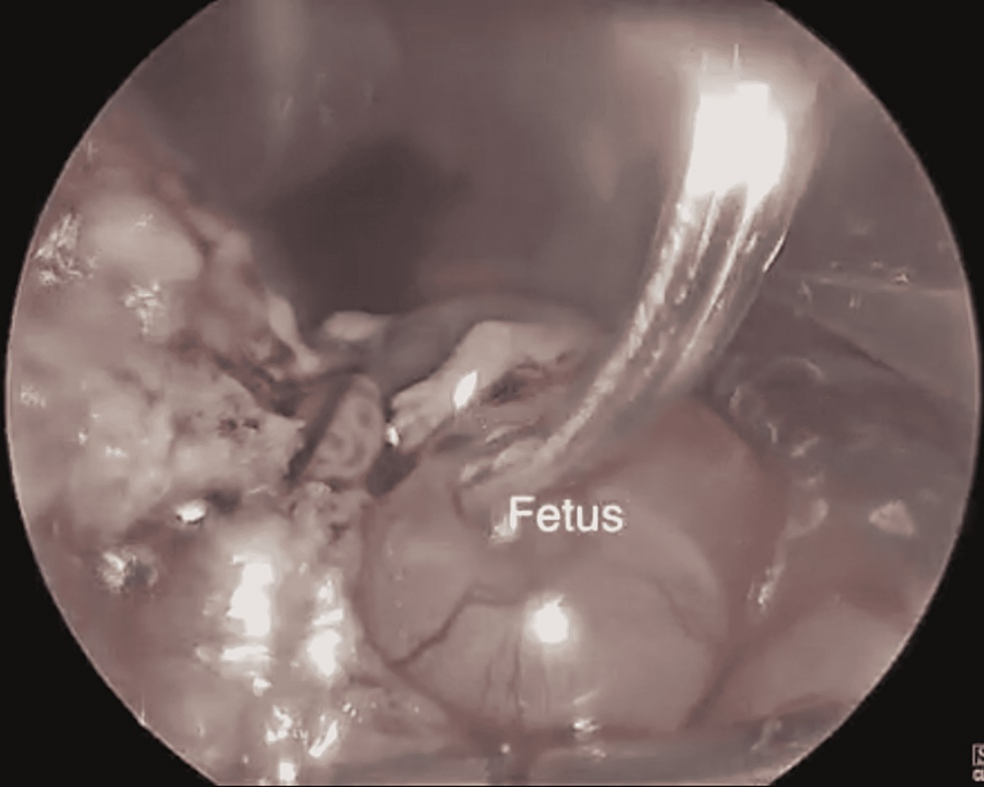
A viable 10-week foetus can be seen laparoscopically

The patient then underwent laparoscopic surgery. Four ports were used, an umbilical port, a suprapubic port, and two lateral ports near the left and right iliac fossae. Once visualized findings were as follows: a non-communicating intact left rudimentary horn pregnancy attached to the uterus with a thick fibrous band 2.5 cm in length and 1.5 cm in width (Figure 3). Laparoscopic excision of both the rudimentary horn and the attached tube was carried out using bipolar diathermy and scissor. The rudimentary horn, the foetus (Figure 4), and the tube were then excised using an endobag after slicing the specimen into small pieces. The patient had minimal blood loss during the surgical procedure and was dismissed home the following day. The patient did well during her follow-up visits and had a smooth recovery.

## Discussion

Mullerian duct anomalies (MDAs) are rare conditions, with a reported incidence of 0.1- 3% in the general population; 2-8% amongst infertile women, and 5-30% amongst women with a history of miscarriage [6,7]. Furthermore, unicornuate uteri make up 2.4-13% of these MDAs [2]. Most unicornuate uteri present with a rudimentary horn and the majority are non-communicating. Rudimentary horns arise as a result of the arrested development of one of the Mullerian ducts [4]. Patients with MDAs are also at an increased risk of renal abnormalities and thus must be investigated for them [8]. Ectopic pregnancy in a rudimentary horn is extremely rare with an incidence being 1 in 76,000 - 150,000 pregnancies [1]. This occurs as a result of transperitoneal migration of the spermatozoa or fertilized ovum from the contralateral tube [2,5]. In the case presented here, the patient had an ectopic pregnancy in a left non-communicating rudimentary horn, that was managed laparoscopically.

Early detection of rudimentary horn pregnancy is crucial, as the risk of uterine rupture is estimated to be 80-90% by the second trimester, which could result in high morbidity and mortality [3]. A high index of suspicion is vital since the majority of cases will present late with massive hemoperitoneum [5]. The ultrasound sensitivity for detection has been reported to be between 29-33%, and an MRI could confirm the diagnosis [9]. Some criteria have also been proposed to help diagnose RHP such as a pseudo pattern of asymmetrical bicornuate uterus, absent visual continuity between cervical canal and lumen of pregnant horn, the presence of myometrium surrounding the gestational sac [10], hypervascularity of the placenta and the presence of an empty uterus with separate gestational sac [3]. A high index of suspicion drew our attention to the possible diagnosis of an RHP in our patient case, specifically the presence of myometrium surrounding the gestational sac and the presence of an empty uterus with a separate gestational sac, as we explained earlier in the case description.

Traditionally, according to reported cases, RHP was treated using laparotomy to excise the rudimentary horn and the ipsilateral fallopian tube, however, with the advancement of surgical innovations and surgical expertise, the laparoscopic technique has become more favorable due to earlier patient recovery [2]. Laparoscopy is especially favorable in cases like our patient where the RHP was diagnosed during the first trimester, but it is worth mentioning that with modern techniques the laparoscopic method has even been utilized for patients in the second trimester [3]. It is accepted practice for some obstetric surgeons to initially use a medical protocol like methotrexate and potassium chloride to terminate the pregnancy first and then perform surgery to excise the rudimentary horn and ipsilateral fallopian tube to prevent future recurrence of ectopic pregnancy [1,9]. However, in our case scenario, we elected to choose the surgical option from the beginning to avoid the risk of waiting and getting untoward outcomes. The patient did well on this line of management and had a smooth post-operative recovery. There have also been reports recommending immediate surgical intervention in unruptured RHPs once diagnosed to ensure the prevention of catastrophic hemoperitoneum [5].

## Conclusions

RHP is a rare entity, requires a high index of suspicion, and can lead to uterine rupture if left untreated. As a result, it is important to be familiar with the proposed ultrasound criterion in the literature that can lead to earlier detection of RHPs. In addition, an MRI can improve accuracy. Due to the earlier diagnosis, we were able to successfully use laparoscopic surgical repair as a treatment modality instead of the traditional laparotomy.
